# The utility of diagnostic tests in the detection and prediction of glucose intolerance in the early and late postpartum period in women after gestational diabetes: a longitudinal cohort study

**DOI:** 10.1186/s13098-021-00650-7

**Published:** 2021-03-17

**Authors:** Dan Yedu Quansah, Justine Gross, Richard Mbundu-Ilunga, Jardena J. Puder

**Affiliations:** 1 Obstetric Service, Department Woman-Mother-Child, Lausanne University Hospital, Lausanne Switzerland Avenue de la Sallaz, CH-1011 Lausanne, Switzerland; 2 Service of Endocrinology, Diabetes and Metabolism, Department of Medicine, Lausanne University Hospital, Lausanne, Switzerland

**Keywords:** Gestational diabetes, Diagnosis, Sensitivity, Glucose-intolerance, Postpartum, Positive predictive value

## Abstract

**Background:**

Due to diverging international recommendations, the unclear role of HbA1c and the lack of longitudinal data, we investigated the accuracy of diagnostic tests in the early and late postpartum in women with gestational diabetes (GDM) especially to predict future glucose-intolerance.

**Methods:**

This longitudinal cohort included 967 women with GDM from 2011 to 2020. A 75-g oGTT and HbA1c were performed at 4–12 weeks (early) postpartum. FPG and HbA1c were measured at 1 and 3-year (late) postpartum. ADA criteria were used as gold standards. At all time-points (4–12 weeks, 1-year and 3-year postpartum) women with diabetes and prediabetes were grouped together and referred to as glucose-intolerant, because at most 3% of the entire cohort population had diabetes at any time-point.

**Results:**

The prevalence of glucose-intolerance in the early postpartum was higher using FPG and HbA1c (27.5%) than oGTT criteria (18.2%). Only 48–80% of women diagnosed with glucose-intolerance in the early postpartum actually remained intolerant. This was especially low when FPG or oGTT were combined with HbA1c (1-year: ≤ 62% and 3-years: ≤ 50%). Regardless of the test used, 1/3 of women with initially normal glucose-tolerance became glucose-intolerant in the late postpartum. HbA1c was unrelated to iron status/intake, remained stable throughout, but poorly predicted future glucose-intolerance. In the longitudinal analyses, all diagnostic tests in the early postpartum showed acceptable specificities (74–96%) but poor sensitivities (all < 38%) to predict glucose-intolerance after only 10-months. At 1-year postpartum however, the combination of FPG and HbA1c could best predict glucose-intolerance 2-years later.

**Conclusions:**

Combining FPG with HbA1c at 1-year postpartum represents a reliable choice to predict future glucose-intolerance. Given the poor prediction of tests including oGTT in the early postpartum, focus should rather be on continuous long-term screening.

**Supplementary Information:**

The online version contains supplementary material available at 10.1186/s13098-021-00650-7.

## Introduction

Gestational diabetes mellitus (GDM) is an independent risk factor of prediabetes and future diabetes [1]. Clinical guidelines recommend re-evaluation for prediabetes or persistent diabetes in the postpartum period in these women [2–4]. The American Diabetes Association (ADA) recommends 75-g oral glucose tolerance test (oGTT) with non-pregnancy diagnostic criteria to identify prediabetes or diabetes at 4–12 weeks postpartum [4, 5], while the National Institute for Health and Care Excellence (NICE) recommends fasting plasma glucose (FPG) at 6–12 weeks, or an HbA1c test after 13 weeks if FPG test is not possible [3]. In order to reduce the long-term maternal risk of diabetes, the ADA recommends a regular postpartum follow-up or testing at 1–3 years interval after an initially normal oGTT results in the early postpartum, whereas the NICE recommends annual HbA1c testing [1, 3].

Despite these recommendations, adherence to postpartum glucose testing is mostly under 50% due to the inconvenience of glucose testing or of performing an oGTT [6, 7]. In 2010 and 2011, in order to simply glucose testing, the ADA and the world health organization (WHO) respectively added the use of HbA1c [8, 9] as an alternative to FPG and oGTT for the diagnosis of diabetes (HbA1c ≥ 6.5%) for the general population. HbA1c has several advantages over FPG and oGTT including its greater convenience (no need for fasting) [10–12], but the ADA does not recommend HbA1c before 1-year postpartum due to fluctuations, blood loss during delivery and changes in iron deficiency [1]. Iron deficiency is common during pregnancy and can lead to increased HbA1c levels, while iron intake/supplementation can decrease HbA1c levels [13–15]. Both could influence the use of HbA1c as a diagnostic test.

Few studies have compared the use of glucose and/or HbA1c tests to oGTT in detecting postpartum glucose intolerance (GI) in women with GDM [5, 16–22]. They were all cross-sectional and their results are controversial [5, 16–22]. In these, HbA1c alone in the early or in the late postpartum was not sufficiently sensitive to detect abnormal glucose tolerance compared to oGTT [16–18]. However, in some studies the combination of HbA1c with FPG was more sensitive [5, 22]. It remains thus unclear, which diagnostic test is most appropriate in the early postpartum to define GI. In general and especially in the light of the COVID-19 pandemic, there is a need to evaluate simplified diagnostic methods in order to reduce person-to person-contact.

Even though some studies have investigated the predictive factors of early and late postpartum glucose intolerance in women with previous GDM [23, 24], no study has investigated the utility of diagnostic tests in the early postpartum to predict future GI. The early postpartum is a time period where most women still show normal glucose tolerance or have prediabetes. Information concerning the characteristics and trajectory of HbA1c in predicting GI in women with prior GDM in this time period is also limited [5] and its role within the first year postpartum remains controversial [1, 3]. To assist preventive strategies, investigating the accuracy and suitability of several diagnostic tests at the early postpartum in predicting future GI could help to clarify and simplify testing procedures, augment postpartum testing adherence and preserve resources. It could also help in the early identification and stratification of both low and high-risk women for timely and targeted postpartum intervention. We conducted this study to assess the prevalence of GI, the accuracy of diagnostic tests in detecting GI in the early and late postpartum and their capacity to predict future GI in a large ethnically diverse clinical cohort of women with GDM.

## Methods

### Study participants

**Fig. 1:**
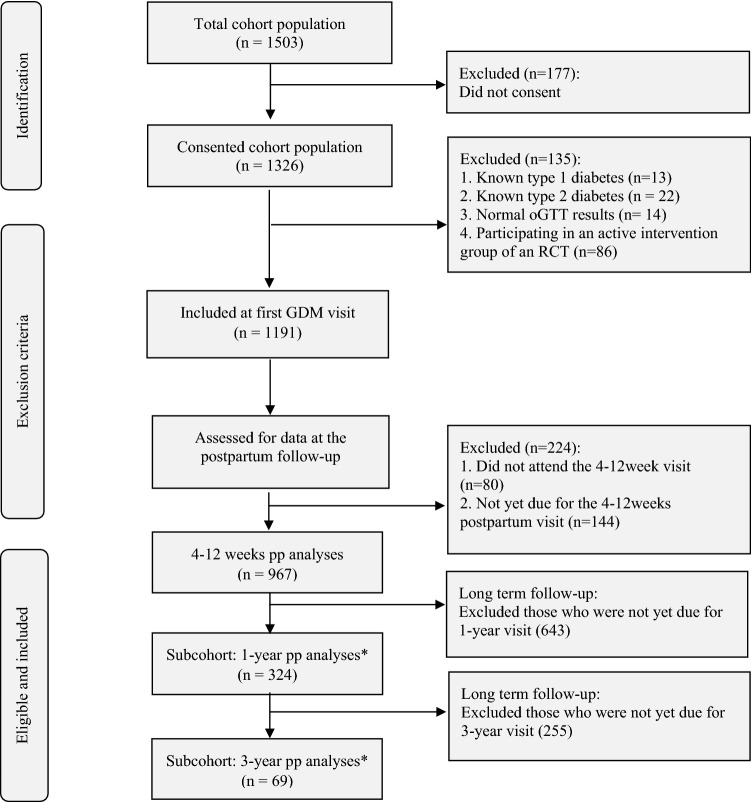
Flow chart describing the selection of study participants. * Subcohort of n = 324 had 1-year data and n = 69 had 3-year data. *pp* postpartum, *GDM* gestational diabetes mellitus, *oGTT* oral glucose tolerance test, *RCT* randomized controlled trial

This prospective clinical longitudinal cohort study followed women with GDM during pregnancy up to 3-years postpartum at the Diabetes and Pregnancy Unit of the Lausanne University Hospital between 2011 and 2020 [25, 26]. The Human Research Ethics Committee of the Canton de Vaud (326/15) approved the study protocol. Pregnant women who diagnosed with GDM at 24–32 weeks gestational age and consented were included. Out of a total cohort population of 1503 women, 1326 women consented to participate representing a response rate of 88.2%. Out of this consented population, we first excluded those who had normal prepartum oGTT results (n = 14), with known type-1 diabetes (n = 13), type-2 diabetes (n = 22) and those who were in the intervention group of an ongoing intervention trial (n = 86) [27]. We also excluded those who did not attend the scheduled 4–12 weeks postpartum follow-up visit (n = 80) or those who were not due to attend (n = 144). Therefore, 967 women were included in the final analyses. Women followed in our unit were seen during pregnancy, assessed at 4–12 weeks postpartum and reassessed at 1-year and 3-year postpartum. Figure 1 shows the details of participants’ selection. They were followed based on the ADA and Endocrine Society recommendation [4, 28] with a special focus on lifestyle counseling and was at least once seen by a dietician. The postpartum visits included the discussion of the laboratory results, assessment of the overall medical situation and lifestyle counseling. The 967 women also included a subgroup population of women (n = 90) who were the control group (CON) of an ongoing intervention trial and had additional data. Their data is shown in Additional file 1: Table S1–S4.

### GDM diagnosis

All women involved in this ongoing clinical cohort were diagnosed with GDM at 24–32 weeks based on the International Association of Diabetes and Pregnancy Study Groups (IADPSG) and the American Diabetes Association (ADA) guidelines [29, 30]. GDM diagnosis was made if one of the following criteria were met during a 75-g oGTT: FPG ≥ 5.1 mmol/l, 1-h glucose ≥ 10.0 mmol/L, or 2-h glucose ≥ 8.5 mmol/l [29, 30].

### Measures

#### Measures of glycemic control

We measured HbA1c at the first GDM visit using the chemical photometric method (conjugation with boronate; Afinion®). In the postpartum visits, HbA1c was assessed using a High Performance Liquid Chromatography method (HPLC) [31]. This method is traceable to the International Federation of Clinical Chemistry and Laboratory Medicine [31] and the Diabetes Control and Complications Trial reference methods. The chemical photometric method (Afinion® analyzer) has similar accuracy and precision with the HPLC method [32]. In the early (4–12 weeks) postpartum visit, a 75-g oGTT was performed to measure FPG and 2-h glucose and in the late (1-year and 3-year) postpartum visits, we measured FPG only. We measured glucose and ferritin from venous blood collected in S-Monovette® tubes containing sodium fluoride and lithium-heparinate respectively. Data regarding iron intake were systematically taken from participants medical charts. Low iron status in the postpartum was defined as serum ferritin ≤ 50 ug/l [33, 34].

In the early (4–12 weeks) postpartum, we used 75-g oGTT criteria as the gold standard, as recommended by the ADA: glucose tolerance status was defined as normal (FPG < 5.6 mmol/l or 2-h glucose < 7.8 mmol/l) vs pathologic (FPG ≥ 5.6 mmol/l or 2-h glucose ≥ 7.8 mmol/l) based on the oGTT results [30]. At 1-year and 3-year postpartum, we used FPG and HbA1c criteria as the gold standard: glucose tolerance was defined as normal (FPG < 5.6 mmol/l or HbA1c < 5.7%) vs pathologic (FPG ≥ 5.6 mmol/l or HbA1c ≥ 5.7%) based on FPG and HbA1c [4, 30]. At all the time points, (6–8 weeks, 1-year and 3-yearpostpartum) GI included women with prediabetes and few women with diabetes. At any time point, at most ≤ 3% of women in the entire cohort had diabetes. This included 2.3% (22/967) women with diabetes at the 4–12 weeks postpartum, 3% (10/324) at 1-year postpartum and 1.4% (1/69) at the 3-year postpartum. Due to the low numbers of the women concerned, we grouped the women with diabetes with the GI and therefore did not use the term prediabetes for this entire group. In the CON subgroup population, we performed a 75-g oGTT at both 4–12 weeks and at 1-year postpartum. GI was defined by oGTT values at 4–12 weeks and by both oGTT and HbA1c at 1-year postpartum [4, 30] (see Additional file 1: Tables S1 and S2). In this subgroup population, one woman who had diabetes at 4–12 weeks and two women who had diabetes at 1-year postpartum were included in the GI group.

In our clinic, women who develop prediabetes at 4–12 weeks postpartum attend a follow-up visit at 3–6 months postpartum and are seen again at 1-year and 3-year postpartum. Except for the visit at 3–6 months postpartum, the number of visits throughout this time period are the same as for women without prediabetes. Those who develop diabetes at either time-points are not followed anymore in our clinic but in the diabetes clinic. In this cohort, the incidence of diabetes was low ( ≤ 3% of the entire cohort population, either at 4–12 weeks, 1-year or 3-year postpartum).

#### Socio-demographic, anthropometric measures and medical characteristics

At baseline (24–32 weeks), maternal information on socio-demographic characteristics including age, nationality/ethnic origin (Switzerland, Europe or north America, Africa, Asia/western pacific, Latin America and others), previous history of GDM (yes/no), family history of diabetes (yes/no) and parity (none, one, two and ≥ three) were collected during a structured face-to-face interview. Information on pre-pregnancy weight was obtained from participants’ medical charts or was self-reported if missing (for the 1–2 months before pregnancy). We measured weight to the nearest 0.1 kg in women wearing light clothes and no shoes with a regularly calibrated electronic scale (Seca®) and height to the nearest 0.1 cm with a regularly calibrated Seca® height scale. Body mass index (BMI) was determined as the ratio of weight in kilograms to the square of height in meters (kg/m^2^). We obtained information on glucose lowering medication during pregnancy (yes/no) and on breastfeeding status at 4–12 weeks postpartum (yes/no) during the routine clinical visits.

### Statistical analysis

We performed all statistical analysis with SPSS software version 26 [35]. Demographic and other descriptive variables were presented as either mean (± standard deviation) for continuous variables and percentages (%) for categorical variables (Table 1). An independent t-test was performed between the value of HbA1c according to the presence or not of postpartum low iron status (Ferritin < 50 µg/l) or iron intake at the respective postpartum time-points. We performed a chi-square test to determine and compare the prevalence of GI according to different GI diagnostic classification methods (75-g OGTT at 4–12 weeks, FPG with HbA1c at 1-year and 3-year) in the early and late postpartum. In the CON population, we assessed and compared the prevalence of GI using the following diagnostic classification methods: 75-g oGTT criteria at 4–12 weeks and with 75-g oGTT with HbA1c criteria at the 1-year postpartum. We investigated the accuracy of various diagnostic tests in identifying GI in the early and late postpartum period in cross-sectional and longitudinal analyses (Tables 3 and 5).

**Table 1:** Socio-demographic and medical characteristics of participants according to glucose tolerance categories based on oGTT criteria at 4–12 weeks postpartum

Variable	All (n = 967)	Normal (n = 670)	Pathological (n = 297)	P-value
	Mean ± SD	Mean ± S D	Mean ± SD	
Glucose tolerance defined by oGTT^a^				
Age (years)	33.0 ± 5.58	32.87 ± 5.70	33.32 ± 5.29	0.244
Gestational age at first GDM visit (weeks)	28.61 ± 3.69	29.02 ± 2.91	27.69 ± 4.93	< 0.001
Pre-pregnancy weight (kg)	69.53 ± 15.65	68.86 ± 15.30	71.05 ± 16.35	0.046
Pre-pregnancy BMI (kg/m^2^)	26.0 ± 5.59	25.63 ± 5.47	26.83 ± 5.76	0.002
Fasting glucose at first GDM visit (mmol/l)	5.17 ± 0.77	5.08 ± 0.71	5.35 ± 0.79	< 0.001
1 hr glucose at GDM diagnosis (mmol/l)	9.62 ± 1.87	9.44 ± 1.82	10.06 ± 1.88	< 0.001
2 hr glucose at GDM diagnosis (mmol/l)	7.85 ± 1.83	7.74 ± 1.74	8.11 ± 2.00	0.012
HbA1c at first GDM visit (%)	5.42 ± 0.41	5.32 ± 0.38	5.65 ± 0.41	< 0.001
Gestational age at delivery (weeks)	38.46 ± 3.10	38.54 ± 3.45	38.29 ± 2.08	0.242
Timing of early postpartum testing (weeks)	8.06 ± 5.21	7.94 ± 5.45	8.32 ± 4.63	0.299
Fasting glucose at 4–12 weeks pp (mmol/l)	5.03 ± 0.56	4.86 ± 0.37	5.42 ± 0.70	< 0.001
2 hr glucose at 4–12 weeks pp (mmol/l)	5.53 ± 1.68	5.08 ± 1.13	6.58 ± 2.20	< 0.001
HbA1c at 4–12 weeks pp (%)	5.34 ± 0.41	5.18 ± 0.30	5.70 ± 0.39	< 0.001
Fasting glucose at 1-year pp (mmol/l) (n = 322)	5.45 ± 0.65	5.33 ± 0.50	5.76 ± 0.90	< 0.001
HbA1c at 1-year pp (%) (n = 324)	5.29 ± 0.40	5.23 ± 0.33	5.48 ± 0.52	< 0.001
Fasting glucose at 3-year pp (mmol/l) (n = 69)	5.49 ± 0.55	5.42 ± 0.52	5.67 ± 0.57	0.086
HbA1c at 3-year pp (%) (n = 69)	5.26 ± 0.32	5.20 ± 0.32	5.41 ± 0.28	0.014

Variable	All (n = 967)	Normal (n = 670)	Pathological (n = 297)	P-value
	n (%)	n (%)	n (%)	
Nationality/ethnic origin				
Switzerland	268 (27.7)	199 (29.7)	69 (23.2)	
Europe + North America	317 (32.7)	237 (35.3)	80 (26.9)	0.001
Africa	170 (17.6)	108 (16.2)	61 (20.5)	
Asia + western pacific	138 (14.3)	78 (11.6)	60 (20.2)	
Latin America	43 (4.4)	29 (4.3)	14 (4.7)	
Others	31 (3.3)	19 (2.8)	13 (4.4)	
Parity				
0	439 (45.4)	324 (48.3)	115 (38.7)	0.015
1	316 (32.6)	215 (32.2)	100 (33.7)	
2	136 (14.0)	84 (12.5)	52 (17.5)	
≥ 3	76 (8.0)	47 (7.0)	30 (10.1)	
Previous history of GDM^b^				
Yes/no	70/897 (7.2/92.8)	33/637 (4.9/95.1)	37/260 (12.5/87.5)	< 0.001
Family history of diabetes^c^				
Yes/no	516/451 (53.4/46.6)	355/315 (52.9/47.1)	162/135 (54.5/45.5)	0.637
Glucose-lowering medication use during pregnancy				
Yes/no	477/490 (49.3/50.7)	290/380 (43.2/56.8)	187/110 (63.0/37.0)	< 0.001
Breastfeeding at 4–12 weeks				
Yes/no	787/180 (81.4/18.6)	549/121 (82.0/18.0)	238/39 (80.1/19.9)	0.499

^a^Normal and Pathological glucose tolerance are according to oGTT criteria in the postpartum period. oGTT denotes oral glucose tolerance test; where normal glucose tolerance means FPG < 5.6 mmol/l or 2 hr glucose < 7.8 mmol/l and Pathological glucose tolerance FPG ≥ 5.6 mmol/l or 2 hr glucose ≥ 7.8 mmol/l. HbA1c denotes glycated hemoglobin. pp means postpartum

^b^GDM denotes gestational diabetes mellitus (12.9% (n = 69) of women who were multiparous had previous history of GDM)

^c^Yes consists of those with first-degree relationship of the participant (e.g. mother, father, brother, sister, daughter, son) and those with second-degree kinship with the participant (e.g. grandparents, grandchildren, nephews, niece, half-brother, half-sister). All values are expressed as mean ± SD or % as indicated. n = 967 unless otherwise stated. Chi-square test was used for categorical variables and ANOVA for continuous variables

In the cross-sectional analyses, we assessed the accuracy of different diagnostic tests (HbA1c alone, FPG alone, 2-h glucose alone, and FPG with HbA1c) in the postpartum with 75-g oGTT as the gold standard at the early postpartum and with FPG with HbA1c at the late postpartum. In the longitudinal analyses, we assessed the predictive capacity of the diagnostic tests (but also oGTT and oGTT with HbA1c) at the early postpartum period using FPG with HbA1c as the gold standard in the late postpartum. We repeated these tests (cross-sectional and longitudinal) in the CON population as sensitivity analyses, but also included the oGTT with HbA1c at the 1-year postpartum as the gold standard to see if this yielded different results. For all analyses, we calculated diagnostic accuracy using sensitivity, specificity, positive predictive value (PPV) and negative predictive value (NPV) of all diagnostic tests. All statistical significances were two sided and accepted at p < 0.05.

## Results

Table 1 shows the socio-demographic and medical characteristics of study participants (n = 967) according to glucose tolerance status (based on 75-g oGTT criteria) at the 4–12 weeks (early) postpartum. This multiethnic cohort had a mean age of 33 ± 5.5 years. The pre-pregnancy BMI and weight were 26 ± 5.6 kg/m^2^ and 69.53 ± 15.6 kg respectively. Out of the total population of 967 participants, 33.4% (n = 324) had completed the 1-year visit and 7.1% (n = 69) had completed the 3-year postpartum visit. The low numbers is because the late postpartum follow-up visits were introduced later after the study was started in 2011 (August 2015 for 1-year and June 2018 for 3-year postpartum visits). Figure 1 shows the details of the participants’ selection. An analysis of the clinical characteristics at 4–12 weeks postpartum between the 324 participants with 1-year data and the 643 without data revealed differences in 2 hr glucose, HbA1c and the prevalence of prediabetes between the two groups. Specifically, 25% (81/324) of the participants who had 1-year data had prediabetes at 4–12 weeks compared to 34% (216/643) with no data. We however found no significant differences in any clinical characteristic between the 69 women who had data at 3-years and those with no data. Please refer to Additional file 1: Table S5 and S6 for details.

### Prevalence of GI and accuracy of diagnostic tests to detect GI at different time-points

The prevalence of GI according to different diagnostic tests at the respective postpartum periods is shown in Table 2. In the early postpartum, the prevalence of GI was 18% (176/967) when using oGTT as the gold standard [30]. It ranged between 13–31% according to the other tests used, was highest when using oGTT combined with HbA1c and almost as high when using FPG and HbA1c (27%). In the late postpartum, the prevalence was 40% (130/324) at 1-year and 39% (27/69) at 3-years when using FPG and HbA1c as the gold standard [30]. Thus, depending on the diagnostic test in the early postpartum, the prevalence of GI was 1.5–3 times higher in the late postpartum.

**Table 2:** Prevalence of glucose intolerance according to different diagnostic tests at different postpartum periods in the entire cohort population

Diagnostic test	All n = 967	Normal n = 791 (81.8%)	Pathological n = 176 (18.2%)
At 4–12 weeks postpartum	n (%)	n (%)	n (%)
Glucose tolerance defined by 75-g oGTT^a^			
Fasting glucose (n = 967)			
Normal	838 (86.8)	791 (100)	49 (27.4)
Pathological	129 (13.2)	0 (0)	127 (72.6)
2 hr glucose (n = 956)			
Normal	870 (91.0)	784 (100)	86 (50.0)
Pathological	86 (9.0)	0 (0)	86 (50.0)
HbA1c (n = 960)			
Normal	768 (80.0)	665 (84.7)	103 (58.9)
Pathological	192 (20.0)	120 (15.3)	72 (41.1)
Fasting glucose and HbA1c (n = 967)			
Normal	702 (72.6)	671(84.8)	31 (17.6)
Pathological	265 (27.4)	120 (15.2)	145 (82.4)
oGTT and HbA1c (n = 967)			
Normal	671 (69.4)	671 (84.8)	0 (0)
Pathological	296 (30.6)	120 (15.2)	176 (100)

Diagnostic test	All n = 324	Normal n = 194 (59.9%)	Pathological n = 130 (40.1%)
At 1-year postpartum	n (%)	n (%)	n (%)
Glucose tolerance defined by FPG and HbA1c^b^			
Fasting glucose (n = 322)			
Normal	210 (65.2)	192 (100)	18 (13.8)
Pathological	112 (34.8)	0(0)	112 (86.2)
HbA1c (n = 324)			
Normal	273 (84.3)	194 (100)	79 (60.8)
Pathological	51 (15.7)	0 (0)	51 (39.2)
Fasting glucose and HbA1c (n = 324)			
Normal	194 (59.9)	194 (100)	0 (0)
Pathological	130 (40.1)	0 (0)	130 (100)

Diagnostic test	All n = 69	Normal n = 42 (60.9%)	Pathological n = 27 (39.1%)
At 3-year postpartum	n (%)	n (%)	n (%)
Glucose tolerance defined by FPG and HbA1c^b^			
Fasting glucose (n = 69)			
Normal	44 (63.2)	42 (100)	2 (7.4)
Pathological	25 (36.8)	0 (0)	25 (92.6)
HbA1c (n = 69)			
Normal	61 (89.7)	42 (100)	19 (73.1)
Pathological	8 (10.3)	0 (0)	8 (26.3)
Fasting glucose and HbA1c (n = 69)			
Normal	42 (60.9)	42 (100)	0 (0)
Pathological	27 (39.1)	0 (0)	27 (100)

oGTT denotes oral glucose tolerance test, FPG denotes fasting glucose, HbA1c denotes glycated hemoglobin

^a^Normal glucose tolerance, defined as FPG < 5.6 mmol/l or 2 hr glucose < 7.8 mmol/l; Pathological defined as FPG ≥ 5.6 mmol/l or 2 hr glucose ≥ 7.8 mmol/l. All pathological values were regrouped as glucose intolerance (= pathological glucose tolerance)

^b^Normal glucose tolerance, defined as FPG < 5.6 mmol/l or HbA1c < 5.7%; Pathological, defined as FPG ≥ 5.6 mmol/l or HbA1c ≥ 5.7%. All pathological values were regrouped as glucose intolerance (= pathological glucose tolerance)

Pathological glucose intolerance includes few women with diabetes and this concerns 22 women at 4–12 weeks postpartum, 10 women at 1-year postpartum and 1 woman at 3-year postpartum. Due to the low number of women concerned, we grouped them into pathological glucose intolerance and therefore did not use the term prediabetes

Table 3 shows the performance of diagnostic tests in detecting GI in the early and late postpartum. In the early postpartum, FPG with HbA1c had over 80% sensitivity and specificity and 96% NPV in diagnosing GI. FPG with HbA1c had better sensitivity but lower specificity and PPV compared to FPG alone. In the late postpartum, FPG alone had a high sensitivity at both 1 and 3-year postpartum (86–92%) with a NPV of over 90%. HbA1c alone on the other hand showed a very poor sensitivity (≤ 41%) at all time-points.

**Table 3:** Detection of glucose intolerance according to diagnostic tests at different postpartum periods (cross-sectional)

Diagnostic test at 4–12 weeks PP	Test characteristics (%)			
	Sensitivity (%)	Specificity (%)	PPV (%)	NPV (%)
At the 4–12 weeks postpartum (n = 967)				
Glucose tolerance defined by 75-g oGTT^a^				
Fasting glucose (mmol/l)	72.6	100	100	94.3
HbA1c (%)	41.1	84.8	37.5	86.6
2 hr glucose (mmol/l)	50.0	100	100	90.1
Fasting glucose and HbA1c	82.4	84.8	54.7	95.6
oGTT and HbA1c	100	84.8	59.5	100

Diagnostic test at 1-year PP	Test characteristics (%)			
	Sensitivity (%)	Specificity (%)	PPV (%)	NPV (%)
At 1-year postpartum (n = 324)				
Glucose tolerance defined by FPG and HbA1c^b^				
Fasting glucose (mmol/l)	86.2	100	100	91.4
HbA1c (%)	39.2	100	100	71.1
Fasting glucose and HbA1c	100	100	100	100

Diagnostic test at 3-year PP	Test characteristics (%)			
	Sensitivity (%)	Specificity (%)	PPV (%)	NPV (%)
Glucose tolerance defined by FPG and HbA1c^b^				
At 3-year postpartum (n = 69)				
Fasting glucose (mmol/l)	92.6	100	100	95.3
HbA1c (%)	26.9	100	100	68.9
Fasting glucose and HbA1c	100	100	100	100

*PPV* positive predictive value, *NPV* negative predictive value, *PP* postpartum, *FPG* fasting glucose, *HbA1c* glycated hemoglobin

^a^oGTT (oral glucose tolerance test) was the gold standard of pathological glucose tolerance at 4–12 weeks postpartum. Normal glucose tolerance, defined as FPG < 5.6 mmol/l or 2 hr glucose < 7.8 mmol/l; Pathological defined as FPG ≥ 5.6 mmol/l or 2 hr glucose ≥ 7.8 mmol/l

^b^FPG and HbA1c was the gold standard of pathological glucose tolerance at 1-year or 3-year postpartum. Normal glucose tolerance defined as FPG < 5.6 mmol/l or HbA1c < 5.7%; Pathological glucose intolerance defined as FPG ≥ 5.6 mmol/l or HbA1c ≥ 5.7%. Pathological glucose intolerance includes few women with diabetes and this concerns 22 women with diabetes at 4–12 weeks postpartum, 10 women at 1-year and 1 woman at 3-year postpartum. Due to the low number of women who had diabetes, we grouped them into pathological glucose intolerance and therefore did not use the term prediabetes

### Impact of low iron status or intake

We did not find any significant mean differences in HbA1c levels between women with/without low iron status or with/without iron intake (all p ≥ 0.32) in the peri-partum period and the late postpartum. Mean HbA1c levels did not significantly differ between 4–12 weeks postpartum (5.3 ± 0.39%, n = 961) and the late postpartum periods (1-year: 5.3 ± 0.4%, n = 324; and 3-year: 5.3 ± 0.3, n = 68; p = 0.91 and 0.3 respectively when compared directly with the early postpartum period). However, the conversion rate from pathological HbA1c in early postpartum to normal HbA1c later on (1-year postpartum) was high (57.7%) for those who had data at both time-points (22/52); p < 0.001). Among these 22 women, the mean HbA1c in the early (5.9 ± 0.2%) and late (6.0 ± 0.5%) postpartum period did not change significantly (p = 0.53). In a subsample of women with pathological HbA1c at 4–12 weeks, mean HbA1c was repeated at 3 (n = 43) and/or 6 (n = 17) months postpartum respectively and did not change (p ≥ 0.24), nor did HbA1c change significantly between these latter two time-points and 1-year postpartum (p ≥ 0.8).

#### Longitudinal changes in GI and accuracy of diagnostic tests to predict future GI

The changes in prevalence of GI according to different diagnostic tests are shown in Table 4. Of women with GI in the early postpartum, 61–80% remained GI at 1-year and 48–75% remained intolerant at 3-year postpartum. This observation was highest for FPG alone, intermediate for oGTT, and lowest for HbA1c combined with either FPG or oGTT as gold standards. For all three gold standards, 33–35% of initially normal glucose tolerant participants converted to GI in the late postpartum.

**Table 4:** Changes in prevalence of glucose intolerance according to diagnostic test and time-period

Diagnostic test at 4–12 weeks pp (total n = 967)^a^ and number of GI at 4–12 weeks pp	Prevalence of GI at 4–12 weeks pp (GI)^b^	Diagnostic test at 1-year pp	Prevalence of GI at 1-year pp (GI)^b^	Diagnostic test at 3-year pp	Prevalence of GI at 3-year pp (still GI)^b^
Glucose-intolerant participants at 4–12 weeks postpartum who remained glucose-intolerant at 1- year and 3-year postpartum					
oGTT (n = 176)	18.2%	FPG & HbA1c	74.5%	FPG & HbA1c	66.7%
FPG & HbA1c (n = 266)	27.5%	FPG & HbA1c	62.2%	FPG & HbA1c	50.0%
FPG alone (n = 127) oGTT & HbA1c (n = 296)	13.2% 30.7%	FPG & HbA1c FPG & HbA1c	80.6% 60.5%	FPG & HbA1c FPG & HbA1c	75.0% 47.6%

Diagnostic test at 4–12 weeks pp (total n = 967)^a^ and number of NGT at 4–12 weeks pp	Prevalence of NGT at 4–12 weeks pp (normal)	Diagnostic test at 1-year pp	Prevalence of GI at 1-year pp (convert GI)	Diagnostic test at 3-year pp	Prevalence of GI at 3-year pp (convert to GI)
Glucose-tolerant participants at 4–12 weeks postpartum who convert to be glucose-intolerant at 1-year or at 3-year postpartum					
oGTT (n = 791)	81.8%	FPG & HbA1c	34.3%	FPG & HbA1c	35.0%
FPG & HbA1c (n = 701)	72.5%	FPG & HbA1c	33.6%	FPG & HbA1c	34.7%
FPG alone (n = 840)	86.8%	FPG & HbA1c	35.1%	FPG & HbA1c	34.4%
oGTT & HbA1c (n = 671)	69.3%	FPG & HbA1c	33.3%	FPG & HbA1c	35.4%

^a^pp denotes postpartum period

^b^GI denotes glucose-intolerant, NGT denotes normal glucose tolerance

oGTT denotes oral glucose tolerance test

*FPG* denotes fasting plasma glucose, *HbA1c* denotes glycated hemoglobin

In brackets refers to the total number of GI or NGT at 4–12 weeks postpartum according to the diagnostic test used

In the upper part of the table, the percentages at 1-year and 3-year postpartum explain how many of initially GI participants remained GI in the postpartum period

In the lower part of the table, the percentages at 1-year and 3-year postpartum explain how many NGT participants converted to GI in the postpartum period

Table 5 shows the performance of diagnostic tests in the early or late postpartum in predicting future GI. All diagnostic tests in the early postpartum had poor sensitivities to predict GI at 1-year and 3-year postpartum (6–38%) although specificity was acceptable (74–96%, lowest for HbA1c and its combinations). Specifically, the highest sensitivities for the prediction of GI at 1-year and 3-years were found for the combinations of oGTT or FPG with HbA1c. The use of diagnostic tests at 1-year postpartum to predict GI 2-years later showed higher sensitivities (up to 78%) compared to the diagnostic test at 4–12 weeks. FPG with HbA1c at 1-year had the highest sensitivity and NPV whereas HbA1c alone had by far the lowest, while specificities and PPV were similar for all diagnostic tests.

**Table 5:** Prediction of future glucose intolerance according to diagnostic tests at different postpartum periods (longitudinal)

	Test characteristics (%)			
Diagnostic test at 4–12 weeks PP	Sensitivity (%)	Specificity (%)	PPV (%)	NPV (%)
Prediction of glucose intolerance 1-year postpartum (n = 324)				
Glucose tolerance defined by FPG and HbA1c^a^				
Fasting glucose (mmol/l)	22.3	96.4	80.6	64.9
HbA1c (%)	21.5	87.5	53.8	62.2
2 hr glucose (mmol/l)	10.1	96.9	68.4	61.6
Fasting glucose and HbA1c	35.4	85.6	62.2	66.4
oGTT	26.9	93.8	74.5	65.7
oGTT and HbA1c	37.7	83.1	59.8	66.7

	Test characteristics (%)			
Diagnostic test at 4–12 weeks PP	Sensitivity (%)	Specificity (%)	PPV (%)	NPV (%)
Prediction of glucose intolerance at 3-year postpartum (n = 69)				
Glucose tolerance defined by FPG and HbA1c^a^				
Fasting glucose (mmol/l)	22.2	95.2	75.0	65.6
HbA1c (%)	18.5	78.6	35.7	60.0
2 hr glucose (mmol/l)	5.8	95.1	54.0	59.1
Fasting glucose and HbA1c	37.0	76.2	50.0	65.3
oGTT	22.2	92.9	66.7	65.0
oGTT and HbA1c	37.0	73.8	47.6	64.6

	Test characteristics (%)			
Diagnostic test at 1-year PP	Sensitivity (%)	Specificity (%)	PPV (%)	NPV (%)
Prediction of glucose intolerance at 3-year postpartum (n = 69)				
Glucose tolerance defined by FPG and HbA1c^a^				
Fasting glucose (mmol/l)	59.3	80.5	66.7	75.0
HbA1c (%)	29.6	90.5	66.7	66.7
Fasting glucose and HbA1c	77.8	76.2	67.7	84.2

*PPV* positive predictive value, *NPV* negative predictive value, *PP* postpartum, *FPG* fasting glucose, *oGTT* oral glucose tolerance test and *HbA1c* glycated hemoglobin

^a^FPG and HbA1c was considered as the gold standard of pathological glucose tolerance at 1-year or 3-year postpartum. Normal glucose tolerance defined as FPG < 5.6 mmol/l or HbA1c < 5.7%; Pathological glucose intolerance defined as FPG ≥ 5.6 mmol/l or HbA1c ≥ 5.7%

Pathological glucose intolerance includes few women with diabetes and this concerns 10 women at 1-year and 1 woman at 3-year postpartum. Due to the low number of women who had diabetes, we grouped them into pathological glucose intolerance and therefore did not use the term prediabetes

In the CON population, 59% (n = 53) had attended the 1-year postpartum visit at the time of this analysis. The prevalence of GI was 11% at 4–12 weeks and 34% at 1-year postpartum, which were slightly lower than in the entire population (Additional file 1: Table S1). Results from the cross-sectional (Additional file 1: Table S2) and longitudinal (Additional file 1: Table S4) analyses regarding sensitivities, specificities, PPV and NPV were similar to the entire population even when the gold standard at 1-year postpartum was oGTT and HbA1c.

## Discussion

Depending on the initial diagnostic test, the prevalence of GI in this clinical cohort of women with GDM was 1.5–3 times higher in the late compared to the early postpartum. Regardless of the test used, 1/3 of originally normal glucose tolerant women later became GI. On the other hand, 48–80% of initially GI women remained intolerant; this was highest when FPG alone or oGTT were used in the early postpartum and lowest for HbA1c alone or its combination with oGTT or FPG. In the cross-sectional analyses, FPG with HbA1c showed the highest sensitivity to diagnose GI, but FPG alone was almost as sensitive. In the longitudinal analyses, all diagnostic tests in the early postpartum including oGTT showed acceptable specificities, but poor sensitivities to predict future GI. The 1-year postpartum tests could more reliably predict GI 2-years later, with the best sensitivities were seen for FPG with HbA1c. At all time-points, HbA1c alone had a poor sensitivity to either diagnose (cross-sectional) or predict (longitudinal) later GI. Mean HbA1c was unrelated to iron status/intake at all time-points, remained stable over time but its conversion rate from pathologic HbA1c in  the early postpartum to normal HbA1c later on and vice versa was high.

Although the choice of testing method in the early postpartum period has a considerable impact on the prevalence of GI, their differences regarding long-term prediction of GI were not flagrant; all showed low sensitivities and high conversion rate to GI later. Long-term screening seems thus an absolute necessity. Based on these results, it would now be most important to put emphasis on long-term follow-up rather than a recommendation of the choice of initial diagnostic test.

A third of women with normal glucose tolerance in the early postpartum who converted to GI in the later postpartum suggests that a normal GI status in the early postpartum should not be reassuring in these women. Furthermore, a third to half of women with initial GI status at 4–12 weeks postpartum reversed to normal glucose tolerance 10 months (1 year postpartum) to 3-years later. The overall prevalence of GI increased substantially after only 10 months even when the same diagnostic criteria were used. This could be due to initial increased insulin sensitivity in the early postpartum, breastfeeding or other causes. Based on the high PPV of FPG or oGTT in the early postpartum and their lower conversion rate to normal glucose tolerance later, more high-risk women could be identified earlier based on these criteria for more intense lifestyle counseling.

In this multiethnic cohort, the prevalence of GI in the early postpartum was especially high when FPG with HbA1c was used compared to oGTT or FPG alone. The prevalence in the late postpartum (40%) was twice as high compared to the 18.4% found by Noctor et al. using the same criteria [20] in a more exclusive Caucasian population. However, it was lower than the 68.5% reported by Kim et al. who studied a small and predominantly obese population, although he included oGTT and not only FPG [19]. In general, the differences in prevalence may be due to the differences in GDM diagnostic criteria, exact late postpartum retesting time periods, BMI and ethnic composition of the cohorts. The latter could also impact on HbA1c [36]. In the cross-sectional analyses, our finding that FPG with HbA1c at 4–12 weeks postpartum had a high sensitivity to diagnose GI is consistent with other cross-sectional studies [5, 19, 22], although this remains controversial [18]. Our 1-year and 3-year postpartum cross-sectional results which suggest that FPG alone or its combination with HbA1c could diagnose GI at both time-points is similar to the data of Picon et al. [16].

According to our longitudinal results, early diagnostic screening had a very poor sensitivity albeit a good specificity to predict later GI irrespective of the classification criteria. The sensitivity was best for the combination of HbA1c with either oGTT or FPG (but still 38% or lower) and 10% or less for the 2-h post-oGTT value alone. Of note, oGTT in combination with HbA1c was not superior to FPG in combination with HbA1c. In contrast to the performance of early screening, FPG and HbA1c at 1-year postpartum had a reliable sensitivity, specificity and especially NPV to predict GI 2-years later. If other studies confirm this observation, it will make risk-stratification at 1-year postpartum especially of low-risk women a valuable option and could save resources. Our results in the late postpartum are in line with two studies in the general population that reported baseline FPG screening as a predictor of diabetes onset 3-years later [37, 38]. Similarly one study identified older adults at high-risk for future diabetes 7-years later by screening with FPG and HbA1c [39].

Regarding HbA1c, our results agree with most cross-sectional studies that showed low sensitivities of HbA1c alone to diagnose GI in the postpartum period [16–18, 20, 40]. In contrast, Katreddy et al. reported a moderate sensitivity (71%) and specificity for HbA1c to detect diabetes but showed lower sensitivity (28%) in detecting overall “impaired glycaemia” (HbA1c ≥ 6%) [41]. To our knowledge, no longitudinal data exist for this population. It is unclear how more than half (58%) of our women with initially pathologic HbA1c in the early postpartum converted to normal HbA1c 1-year later (their mean HbA1c did not change). Importantly, mean HbA1c of women with pathological values did not change between 4 and 12 weeks (average of 8.1 ± 5.2 weeks) and 3 or 6-months postpartum or between 3–6-months and 1-year postpartum. Based on our data the NICE recommendation to wait until 3-months postpartum and the ADA recommendation to wait until 1-year postpartum to perform an HbA1c could be adapted if other studies confirm our results [3]. Interestingly, HbA1c was unrelated to iron deficiency or intake in our cohort. This is inconsistent with a systematic review in which iron deficiency led to an increase in HbA1c levels, albeit during pregnancy and a previous report that suggested that iron intake lowers HbA1c levels [13] as well as blood loss during delivery.

This study has many strengths. Of utmost importance is its novel longitudinal design. It also included a subgroup of women with 1-year oGTT data for sensitivity analysis. Results from the subgroup were similar to the main population, even when oGTT and HbA1c were used as a gold standard in the late postpartum. It is the first to investigate the cross-sectional accuracy and the longitudinal prediction of GI in the postpartum using exhaustive analyses of several clinically used diagnostic tests at different clearly defined time-points rather than comparing one diagnostic test with a gold standard. We used a large and ethnically diverse cohort, which increases external validity and generalizability of our results.

Despite this, evolution to GI and the potential accuracy of the test to predict might differ between various centers, patient populations and countries. As testing in the late postpartum period started only in 2015 (1-year) and 2018 (3-years), the proportion of women followed in the late postpartum were relatively low and their results may not be comparable to the early postpartum. We compared the clinical characteristics of participants at 4–12 weeks postpartum between those with and without 1-year data and found differences in the 2 hr glucose, HbA1c and the prevalence of prediabetes. We did not find any significant differences in any clinical characteristic between women with 3-year data and those with no data. Another limitation is the various influences that take place within 3-years which is independent of the choice of the testing method, including changes in lifestyle or in weight. It is also worthy to note that we used the FPG and HbA1c criteria to define GI status at 1-year and 3-years in the large cohort rather than the OGTT criteria [1]. In the subgroup population however, OGTT alone (and OGTT and HbA1c) criteria were used at 1-year postpartum and the predictive performance of diagnostic tests were similar to that of the cohort. Furthermore, very few women were diagnosed with GI based on 2 hr glucose alone, either at 4–12 weeks postpartum (4.4%) in the entire cohort or at 1-year postpartum (3.8%) in the subgroup. It remains important to assess the predictability of tests in this natural setting to be able to risk-stratify these women. Of note, mean BMI did not change between the early and late postpartum and the prevalence of diabetes in our cohort was relatively low.

## Conclusion

The choice of test to diagnose GI in the early postpartum after GDM depends if the objective is for example to use the diagnosis of GI as an additional motivation for lifestyle changes (using a test with high sensitivity) or to avoid unnecessary anxiety (using a test with high specificity). Although the combination of FPG and HbA1c could be used in the early postpartum, FPG alone had higher PPV and could be used in this time period to specifically select women at higher risk for more intensive lifestyle intervention. As all diagnostic tests used in the early postpartum had a very low sensitivity to predict GI even after 10-months, a focus on long-term screening is more important. Importantly and in contrast to the ADA recommendation, we found no clear advantage of performing oGTT over FPG in the early postpartum period. Thus, feasibility should be an important criteria in the choice of initial early diagnostic testing after GDM. In discordance with the NICE recommendation, we could not confirm the need to postpone HbA1c testing to after 3 months postpartum, as mean HbA1c did not change throughout the first year. However, in view of its low specificity and very low PPV, and high conversion rate to normal glucose tolerance, its use as a diagnostic tool during the 1-year postpartum should only be reserved for selected patients and situations. Taking into consideration our longitudinal data, the combination of FPG with HbA1c seems to be a valid choice in the late postpartum where it reliably predicted GI 2-years later.

## Supplementary Information

**Additional file 1: Table S1.** Prevalence of glucose intolerance according to different diagnostic tests at different postpartum periods in CON participants. Table S2. Detection of glucose intolerance according to diagnostic tests at different postpartum periods (cross-sectional) in CON participants. Table S3. Changes in prevalence of glucose intolerance according to diagnostic tests in CON participants. Table S4. Prediction of 1-year pathological glucose intolerance according to diagnostic tests at 4–12 weeks postpartum period in CON participants (longitudinal) (N=60). Table S5. Clinical characteristics at 4–12 weeks postpartum between participants with and without data for the 1-year postpartum visit. Table S6. Clinical characteristics at 4–12 weeks postpartum between participants with and without data for the 3-year postpartum visit.

## Data Availability

The datasets generated and/or analyzed during the current study are not publicly available [the data used in the article are clinical data from women with gestational diabetes in the Lausanne University hospital which is maintained by the gestational diabetes clinic, and are kept in a secure server] but are available on reasonable request to the corresponding author.

## Abbreviations

IADPSG: Association of Diabetes and Pregnancy Study Groups
FPG: Fasting plasma glucose
GDM: Gestational diabetes mellitus
GI: Glucose intolerance
NICE: National Institute for Health and Care Excellence
oGTT: 75-g oral glucose tolerance test
