# Supplementary material for: The utility of diagnostic tests in the detection and prediction of glucose intolerance in the early and late postpartum period in women after gestational diabetes: a longitudinal cohort study
Source: Diabetol Metab Syndr. 2021 Mar 17;13:31. doi: 10.1186/s13098-021-00650-7 (PMC7966915; doi:10.1186/s13098-021-00650-7)
Supplement: Supplementary file 1 — Additional file 1: Table S1. Prevalence of glucose intolerance according to different diagnostic tests at different postpartum periods in CON participants. Table S2. Detection of glucose intolerance according to diagnostic tests at different postpartum periods (cross-sectional) in CON participants. Table S3. Changes in prevalence of glucose intolerance according to diagnostic tests in CON participants. Table S4. Prediction of 1-year pathological glucose intolerance according to diagnostic tests at 4–12 weeks postpartum period in CON participants (longitudinal) (N=60). Table S5. Clinical characteristics at 4–12 weeks postpartum between participants with and without data for the 1-year postpartum visit. Table S6. Clinical characteristics at 4–12 weeks postpartum between participants with and without data for the 3-year postpartum visit. [file 13098_2021_650_MOESM1_ESM.docx]

**Supplementary tables**

**Table 1:** Prevalence of glucose intolerance according to different diagnostic tests at different postpartum periods in CON participants

|  | **Glucose tolerance defined by 75-g oGTT^a^** | | |
| --- | --- | --- | --- |
| **Diagnostic test** | **All**  **(90)** | **Normal**  **n=80 (88.9%)** | **Pathological**  **n=10 (11.1%)** |
| **At 4-12 weeks postpartum** | n (%) | n (%) | n (%) |
| Fasting glucose (n=90) |  |  |  |
| Normal | 84 (93.3) | 80 (100) | 4 (40.0) |
| Pathological | 6 (6.7) | 0 (0) | 6 (60.0) |
| 2hr glucose (n=90) |  |  |  |
| Normal | 84 (93.3) | 80 (100) | 4 (40.0) |
| Pathological | 6 (6.7) | 0 (0) | 6 (60.0) |
| HbA1c (n=90) |  |  |  |
| Normal | 78 (87.6) | 72 (89.9) | 7 (70.0) |
| Pathological | 12 (12.4) | 8 (10.1) | 3 (30.0) |
| Fasting glucose and HbA1c (n=90) |  |  |  |
| Normal | 75 (83.3) | 72 (90.0) | 3 (30.0) |
| Pathological | 15 (16.7) | 8 (10.0) | 7 (70.0) |
| oGTT and HbA1c n=90) |  |  |  |
| Normal | 72 (80.0) | 72 (90.0) | 0 (0) |
| Pathological | 18 (20.0) | 8 (10.0) | 10 (100.0) |
|  | **Glucose tolerance defined by 75-g oGTT and HbA1c^b^** | | |
| **Diagnostic test** | **All**  **n=53** | **Normal**  **n=35 (66.0%)** | **Pathological**  **n=18 (34.0 %)** |
| **At 1-year postpartum** | n (%) | n (%) | n (%) |
| Fasting glucose (n=53) |  |  |  |
| Normal | 37 (69.8) | 35 (100) | 2 (11.1) |
| Pathological | 16 (30.2) | 0 (0) | 16 (88.9) |
| 2hr glucose (n=53) |  |  |  |
| Normal | 51 (96.2) | 35 (100) | 13 (72.2) |
| Pathological | 2 (3.8) | 0 (0) | 5 (27.8) |
| HbA1c (n=53) |  |  |  |
| Normal | 50 (94.3) | 35 (100) | 15 (83.3) |
| Pathological | 3 (5.7) | 0 (0) | 3 (16.7) |
| Fasting glucose and HbA1c (n=53) |  |  |  |
| Normal | 36 (67.9) | 35 (100) | 1 (5.6) |
| Pathological | 17 (32.1) | 0 (0) | 17 (94.4) |
| oGTT and HbA1c (n=53) |  |  |  |
| Normal | 35 (66.0) | 35 (100.0) | 0 (0) |
| Pathological | 18 (34.0) | 0 (0) | 18 (100.0) |

oGTT denotes oral glucose tolerance test, FPG denotes fasting glucose, HbA1c denotes glycated hemoglobin

^a^Normal glucose tolerance, defined as FPG <5.6mmol/l or 2hr glucose <7.8mmol/l; Pathological defined as FPG ≥5.6mmol/l or 2hr glucose ≥7.8mmol/l.

^b^Normal glucose tolerance, defined as FPG <5.6mmol/l or 2hr glucose <7.8mmol/l or HbA1c <5.7%; Pathological defined as FPG ≥5.6mmol/l or 2hr glucose ≥7.8mmol/l or HbA1c ≥5.7%.

Pathological glucose intolerance includes few women with diabetes and this concern 1 women at 4-12 weeks postpartum and 2 women at 1-year postpartum. Due to the low number of women who had diabetes, we grouped them into pathological glucose intolerance and therefore did not use the term prediabetes

**Table 2:** Detection of glucose intolerance according to diagnostic tests at different postpartum periods (cross-sectional) in CON participants

| **At 4-12 weeks postpartum (n=9**0) | | | | |
| --- | --- | --- | --- | --- |
| Glucose tolerance defined by 75-g oGTT^a^ | **Test characteristics (%)** | | | |
| **Diagnostic test at 4-12 weeks PP** | **Sensitivity (%)** | **Specificity (%)** | **PPV (%)** | **NPV (%)** |
| Fasting glucose (mmol/l) | 60.0 | 100 | 100 | 95.2 |
| HbA1c (%) | 30.0 | 89.9 | 27.3 | 91.0 |
| 2hr glucose (mmol/l) | 60.0 | 100 | 100 | 95.2 |
| Fasting glucose and HbA1c | 70.0 | 90.0 | 46.7 | 96.0 |
| oGTT and HbA1c | 100 | 90.0 | 55.6 | 100 |
| **At 1-year postpartum (n=53)** | |  |  |  |
| Glucose tolerance defined by 75-g oGTT and HbA1c^b^ | **Test characteristics (%)** | | | |
| **Diagnostic test at 1-year PP** | **Sensitivity (%)** | **Specificity (%)** | **PPV (%)** | **NPV (%)** |
| Fasting glucose (mmol/l) | 88.9 | 100 | 100 | 94.6 |
| HbA1c (%) | 16.7 | 100 | 100 | 70.0 |
| 2hr glucose (mmol/l) | 13.3 | 100 | 100 | 74.5 |
| Fasting glucose and HbA1c | 94.4 | 100 | 100 | 97.2 |
| Fasting and 2hr glucose | 88.9 | 100 | 100 | 94.9 |
| oGTT and HbA1c | 100 | 100 | 100 | 100 |
| **At 1-year postpartum (n=53)** | |  |  |  |
| Glucose tolerance defined by 75-g oGTT^a^ | **Test characteristics (%)** | | | |
| **Diagnostic test at 1-year PP** | **Sensitivity (%)** | **Specificity (%)** | **PPV (%)** | **NPV (%)** |
| Fasting glucose (mmol/l) | 94.1 | 100 | 100 | 97.3 |
| HbA1c (%) | 11.8 | 97.2 | 66.7 | 70.0 |
| 2hr glucose (mmol/l) | 14.3 | 100 | 100 | 76.5 |
| Fasting glucose and HbA1c | 94.1 | 97.2 | 94.1 | 97.2 |
| Fasting and 2hr glucose | 100 | 100 | 100 | 100 |
| oGTT and HbA1c | 100 | 94.9 | 88.9 | 100 |

PPV denotes positive predictive value, NPV denotes negative predictive value, PP denotes postpartum, FPG denotes fasting glucose and HbA1c denotes glycated hemoglobin.

^a^oGTT (oral glucose tolerance test) was the gold standard of pathological glucose tolerance at 4-12 weeks postpartum. Normal glucose tolerance, defined as FPG <5.6mmol/l or 2hr glucose <7.8mmol/l; Pathological defined as FPG ≥5.6mmol/l or 2hr glucose ≥7.8mmol/l.

^b^oGTT and HbA1c was the gold standard of pathological glucose tolerance at 1-year postpartum. Normal glucose tolerance defined as FPG <5.6mmol/l or 2hr glucose or HbA1c <5.7%; Pathological glucose intolerance defined as FPG ≥5.6mmol/l or 2hr glucose ≥7.8mmol/l or HbA1c ≥5.7%. Pathological glucose intolerance includes few women with diabetes and this concern 1 woman at 4-12 weeks postpartum and 2 women at 1-year postpartum. Due to the low number of women concerned, we grouped them into pathological glucose intolerance and therefore did not use the term prediabetes

**Table 3:** Changes in prevalence of glucose intolerance according to diagnostic tests in CON participants

| **Diagnostic test at 4-12 weeks pp (total n=90)** | **Prevalence**  **(GI)** | **Diagnostic test 1-year**  **pp** | **Prevalence**  **(still GI)** |
| --- | --- | --- | --- |
| 75-g oGTT (n=10) | 11.1% | FPG & HbA1c  75-g oGTT & HbA1c | 75%  60% |
| FPG & HbA1c (n=15) | 16.7% | FPG & HbA1c  75-g oGTT & HbA1c | 57.1%  40% |
| FPG alone (n=6) | 6.7% | FPG & HbA1c  75-g oGTT & HbA1c | 75%  75% |
| oGTT and HbA1c (n=18) | 20.0% | FPG & HbA1c  75-g oGTT & HbA1c | 57.1%  36.4% |

pp denotes postpartum period; GI denotes glucose-intolerant; oGTT denotes oral glucose tolerance test; FPG denotes fasting plasma glucose; HbA1c denotes glycated hemoglobin

**Table 4:** Prediction of 1-year pathological glucose intolerance according to diagnostic tests at 4-12 weeks postpartum period in CON participants (longitudinal) (N=60)

| **Glucose tolerance defined by 75-g oGTT and HbA1c^a^** | **Test characteristics (%)** | | | |
| --- | --- | --- | --- | --- |
| **Diagnostic test at 4-12 weeks PP** | **Sensitivity (%)** | **Specificity (%)** | **PPV**  **(%)** | **NPV (%)** |
| Fasting glucose (mmol/l) | 16.7 | 97.6 | 75.0 | 73.2 |
| HbA1c (%) | 11.1 | 85.4 | 25.0 | 68.6 |
| 2hr glucose (mmol/l) | 5.6 | 97.6 | 50.0 | 70.7 |
| Fasting glucose and HbA1c  Fasting and 2hr glucose | 22.2  16.7 | 85.7  95.2 | 40.0  60.0 | 72.0  72.7 |
| OGTT and HbA1c | 22.2 | 83.3 | 36.4 | 71.4 |

PPV denotes positive predictive value, NPV denotes negative predictive value, PP denotes postpartum, FPG denotes fasting glucose and HbA1c denotes glycated hemoglobin.

^a^FPG and HbA1c was considered as the gold standard of pathological glucose tolerance at 1 or 3-year postpartum. Normal glucose tolerance defined as FPG <5.6mmol/l or HbA1c <5.7%; Pathological glucose intolerance defined as FPG ≥5.6mmol/l or HbA1c ≥5.7%. Pathological glucose intolerance includes few women with diabetes and this concern 1 woman at 4-12 weeks postpartum and 2 women at 1-year postpartum. Due to the low number of women concerned, we grouped them into pathological glucose intolerance and therefore did not use the term prediabetes

Supplementary Table 5: Clinical characteristics at 4-12 weeks postpartum between participants with and without data for the 1-year postpartum visit.

|  | **Participants with data for the 1-year postpartum visit** | | |  |
| --- | --- | --- | --- | --- |
| **Variable** | **All**  **(n=967)** | **Yes**  **(n=324)** | **No***  **(n=643)** | **P-value** |
|  | **Mean ± SD** | **Mean ±S D** | **Mean ± SD** |  |
| Age (years) | 33.0±5.58 | 33.16±5.73 | 32.92±5.51 | 0.534 |
| Gestational age at delivery (weeks) | 38.46±3.10 | 38.74±1.89 | 38.32±3.55 | **0.045** |
| BMI at 6-8 weeks (kg/m^2^) | 27.71±5.43 | 27.60±5.34 | 27.76±5.48 | 0.654 |
| Weight at 6-8 weeks (kg) | 74.10±15.28 | 73.76±14.66 | 74.28±15.60 | 0.621 |
| Weight retention (kg) | 4.61±5.85 | 4.61±6.25 | 4.61±5.85 | 0.987 |
| Fasting glucose at 4-12 weeks pp (mmol/l) | 5.03±0.56 | 4.99±0.48 | 5.05±0.59 | 0.140 |
| 2hr glucose at 4-12 weeks pp (mmol/l) | 5.53±1.68 | 5.26±1.48 | 5.67±1.76 | **0.001** |
| HbA1c at 4-12 weeks pp (%) | 5.34±0.41 | 5.30±0.39 | 5.37±0.42 | **0.011** |
| Prediabetes at 6-8 weeks | **n (%)** | **n (%)** | **n (%)** |  |
| Yes | 297 (30.7) | 81 (24.9) | 216 (33.6) | **0.006** |
| No | 670 (69.3) | 243 (75.1) | 427 (66.4) |  |

*No means yet to attend postpartum visit

All values are expressed as mean± SD or % as indicated. Chi-square test was used for categorical variables and ANOVA for continuous variables.

Supplementary Table 6 Clinical characteristics at 4-12 weeks postpartum between participants with and without data for the 3-year postpartum visit.

|  | **Participants with data for the 1-year postpartum visit** | | |  |
| --- | --- | --- | --- | --- |
| **Variable** | **All**  **(n=967)** | **Yes**  **(n=69)** | **No***  **(n=898)** | **P-value** |
|  | **Mean ± SD** | **Mean ±S D** | **Mean ± SD** |  |
| Age (years) | 33.0±5.58 | 32.83±4.77 | 33.02±5.64 | 0.776 |
| Gestational age at delivery (weeks) | 38.46±3.10 | 38.81±1.88 | 38.43±3.18 | 0.312 |
| BMI at 6-8 weeks (kg/m^2^) | 27.71±5.43 | 26.80±4.97 | 27.78±5.46 | 0.141 |
| Weight at 6-8 weeks (kg) | 74.10±15.28 | 71.35±14.27 | 74.32±15.35 | 0.112 |
| Weight retention (kg) | 4.61±5.99 | 4.29±5.84 | 4.64±6.00 | 0.635 |
| Fasting glucose at 4-12 weeks pp (mmol/l) | 5.03±0.56 | 4.97±0.52 | 5.03±0.56 | 0.342 |
| 2hr glucose at 4-12 weeks pp (mmol/l) | 5.53±1.68 | 5.28±1.55 | 5.53±1.69 | 0.817 |
| HbA1c at 4-12 weeks pp (%) | 5.34±0.41 | 5.35±0.40 | 5.34±0.41 | 0.860 |
| Prediabetes at 6-8 weeks (n, %) | **n (%)** | **n (%)** | **n (%)** |  |
| Yes | 297 (30.7) | 24 (34.78) | 273 (30.4) | 0.797 |
| No | 670 (69.3) | 45 (65.22) | 625 (69.6) |  |

*No means yet to attend postpartum visit

All values are expressed as mean± SD or % as indicated. Chi-square test was used for categorical variables and ANOVA for continuous variables.
